# Regulation of immune responses by a tumor necrosis factor in pearl oysters: insights from *PmTNF* gene expression and function: Characterization of novel *TNF* in pearl oyster

**DOI:** 10.3724/abbs.2025003

**Published:** 2025-02-27

**Authors:** Yifan Wu, Bidan Liang, Haiying Liang

**Affiliations:** 1 Fisheries College Guangdong Ocean University Zhanjiang 524088 China; 2 Guangdong Provincial Key Laboratory of Aquatic Animal Disease Control and Healthy Culture Zhanjiang 524088 China

**Keywords:** *Pinctada fucata martensii*, tumor necrosis factor, NF-κB, RNA interference, nucleus insertion

## Abstract

Tumor necrosis factor (TNF) is a multifunctional cytokine that regulates cellular processes such as inflammation, apoptosis, differentiation, and proliferation and activates various functions of the immune system. This article reports the discovery and characterization of a novel tumor necrosis factor gene in the pearl oyster *Pinctada fucata martensii*, which is named *PmTNF*. The deduced PmTNF protein sequence displays the typical structural characteristics of a TNF domain, and phylogenetic analysis of the sequences of PmTNF and its putative orthologs shows that they conform to the current taxonomy. Analysis of *PmTNF* mRNA expression via real-time PCR reveals its constitutive expression in all the examined tissues, with the highest expression in the gills. Furthermore, *PmTNF* expression in the gills varies upon exposure to pathogen-derived stimuli, with modest upregulation in response to lipopolysaccharides, but with significant downregulation in response to polyinosinic:polycytidylic acid. Nucleus insertion surgery induces an increase in *PmTNF* mRNA level in the gills at 12 h postoperation. Knocking down *PmTNF* through RNA interference significantly inhibits the expressions of immune-related genes in the NF-κB signaling pathway in the gills by 24 h ( *P* < 0.05). The function of *PmTNF* is further characterized by studying the activity of an engineered recombinant PmTNF protein (rPmTNF) *in vivo*. Upon nuclear insertion, treatment with rPmTNF for 6 h upregulates several genes in the NF-κB pathway. Similarly, rPmTNF increases the activities of the antioxidant enzymes, including superoxide dismutase, glutathione and peroxidase, which reflect the total antioxidant capacity. Collectively, these results indicate that *PmTNF* participates in pearl oyster immunity by modulating the NF-κB pathway and activating the antioxidant defense system.

## Introduction

TNF-α is a key member of the TNF ligand family that exists as membrane-bound and soluble cytokines, and it is released from the cell surface after proteolytic cleavage, mainly by metalloproteinases. Different metalloproteinases can be induced by various stimuli, and the resulting soluble TNF forms possibly play distinct physiological roles [1]. Members of the tumor necrosis factor (TNF) family and its closely related receptors constitute potent cytokine axes that are implicated in proinflammatory and immunological responses. They are involved in several crucial biological processes, such as cytotoxicity and antiviral activities [2] .

Since TNF cDNA was first identified in humans in 1984 [3]. Nineteen TNF members have been characterized in mammals in recent decades [4] and are classified into two major types: TNF-α (also called cachectin) and TNF-β (also called lymphotoxin) [5]. The majority of TNF members are expressed by immune cells, such as monocytes, macrophages, T lymphocytes, and natural killer cells [6]. In fish, two types of TNF-α, termed *shTNF-α1* and *shTNF-α2*, have been identified in snakehead ( *Channa argus*), suggesting that they play important roles in bacterial infection and pathogen clearance [7]. Furthermore, a TNF gene (named *RB-TNFN*) in rock bream ( *Oplegnathus fasciatus*) was cloned, and its functional expression was analyzed, revealing that *RB-TNFN* is highly conserved with TNF family signatures and mediates innate and adaptive immunity [8]. The TNF superfamily and its intricate regulatory processes have been studied extensively in vertebrates, and currently, an expanding array of TNF superfamily members has been discovered in various invertebrates. The initial discovery of an invertebrate TNF ligand (Eiger) isolated from *Drosophila* revealed that it plays a pivotal role in the modulation of extracellular pathogen growth, thereby contributing to the host immune defense mechanisms [9]. A TNF ligand ( *Mj*TNF), which contains a predicted transmembrane region and a TNF homology domain, has been characterized in the marine arthropod kuruma shrimp ( *Marsupenaeus japonicus*). A high expression level of *Mj*TNF was observed *in vivo* after stimulation with lipopolysaccharide [10]. A new member of the TNF superfamily (named *EsTNFSF*) was identified from the Chinese mitten crab ( *Eriocheir sinensis*), which also contains a transmembrane region and a conserved extracellular C-terminal TNF domain [11]. Additionally, several TNF homologs have been identified and characterized in mollusks. The first molluscan TNF-α, *AbTNF-α*, was cloned from disk abalone ( *Haliotis discus discus*) [12], and its mRNA expression in gill tissue was significantly induced by a mixture of pathogenic bacteria and lipopolysaccharide. The transcript level of *ChTNF*, a TNF homolog of oyster ( *Crassostrea hongkongensis*), was significantly increased in blood cells following immune challenge [13]. Two novel TNF genes ( *CgTNF-1* and *CgTNF-2*) have been characterized in pacific oysters ( *Crassostrea gigas*), both of which have been implicated in the immune response. *Cg*TNF-1 is involved in the modulation of immune responses, such as the regulation of antibacterial activity and the activation of immune-related enzymes. *Cg*TNF-2 promotes antibacterial activity by inducing lysozyme activity [ 14, 15]. It was reported that the TNFα of *Sepiella japonica* (named *SjTNFα*) could enhance resistance to bacteria by activating the NF-κB pathway in the nucleus [16]. The proteins encoded by these genes are structurally and functionally similar to their mammalian counterparts, having a TNF homology domain (THD) and being involved in the host immune response.

The pearl oyster *Pinctada fucata martensii* is a marine bivalve with significant economic value in terms of pearl cultivation and is naturally distributed in southern China, Japan, Australia, and Southeast Asia [ 17, 18]. To culture round pearls, nucleus insertion is performed. This technique involves implanting a spherical shell bead, which is enveloped by fragments of the mantle epithelium, into the visceral mass of the receiver oyster [17]. This implantation may cause injury, ultimately leading to bacterial or viral diseases, immune rejection, discharge of the nucleus, and/or death of the host [19]. Therefore, studying pearl oyster innate immunity is crucial for reducing the immune rejection rate and enhancing pearl production by *P*. *f*. *martensii* .

The aim of this study was to identify potential TNF homologs in pearl oysters and investigate their regulation at the molecular level. We report the discovery and cloning of a full-length cDNA corresponding to a TNF homolog in *P*. *f*. *martensii*. which we named *PmTNF*. We characterized the transcriptional pattern of *PmTNF* in oyster tissues and its regulation during nucleus insertion. Furthermore, we tested *PmTNF* function *in vivo*. To explore this functional approach, we compared the regulation of immune-related genes of the NF-κB signaling pathway during the exposure of pearl oysters to pathogen-derived lipopolysaccharides (LPS) and polyinosinic:polycytidylic acid [Poly(I:C)] when *PmTNF* was knocked down via RNA interference or upon the provision of exogenous recombinant PmTNF protein (rPmTNF).

## Materials and Methods

### Experimental design

**Figure 1:**
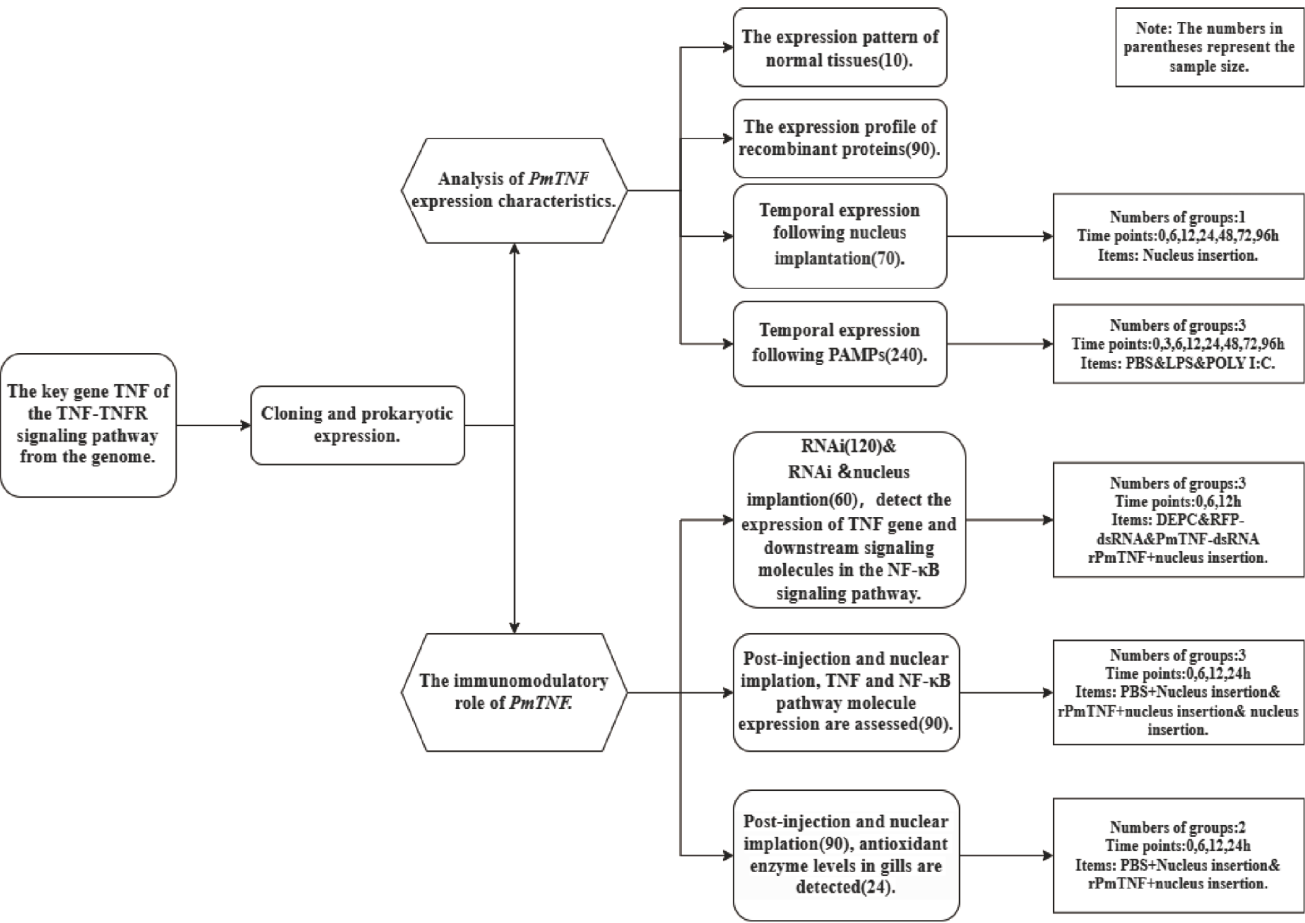
Figure 1 The flowchart of the experimental design Types of designs (such as control and randomization), the number of groups or conditions, and the sample size.

This study aimed to understand the role of *PmTNF* in the TNF-TNFR (tumor necrosis factor receptor) signaling pathway and how it affects immune responses and the expression of antioxidant enzymes. The key gene of the TNF-TNFR signaling pathway, *TNF*, was cloned from the genome and expressed prokaryotically. The expression characteristics of *PmTNF*, along with its immunomodulatory effects, were analyzed. Each experimental step of *PmTNF* involves different experimental groups and time points, as shown in Figure 1.

### Animals

Healthy pearl oysters (approximately 2 years of age) were obtained directly from the Dajing pearl farming base in Xuwen (Zhanjiang, China). Pearl oysters were acclimated in circulating seawater tanks at 25–27°C for five days prior to the initiation of research.

### Cloning and sequence analysis of *PmTNF-α*

Specific primers (see sequences in Table 1) were designed from the partial sequence of the *PmTNF* gene, which was obtained from the transcriptome sequencing of *P*. *f*. *martensii* hemocytes and was predicted to contain a typical TNF domain [20]. A total volume of 10 μL was used for the PCR procedure to amplify the intermediate fragment, comprising 5 μL of Premix Taq, 0.4 μL of template cDNA, 0.4 μL of each primer and 3.8 μL of double-distilled water. The PCR thermal cycling conditions were as follows: denaturation at 95°C for 5 min; 38 cycles of denaturation at 95°C for 30 s, annealing at 55°C for 30 s, and extension at 72°C for 2 min; and a final extension at 72°C for 10 min. The PCR product of the predicted size was extracted from the agarose gel, purified via a DNA fragment recovery kit (Takara Bio, Dalian, China), and subcloned and inserted into the pMD-19 T vector (Takara Bio), after which the ligation mixture was introduced into chemically competent *Escherichia coli* DH5α cells for transformation. The 5′ and 3′ ends of the targeted sequence were acquired via the rapid amplification of cDNA ends (RACE) technique, which employs the SMARTer ^TM^ RACE cDNA Amplification Kit (Takara Bio). The full-length *PmTNF* sequence was spliced via DNAMAN software. The NCBI BLAST program was utilized to search for nucleotide and protein sequences similar to those of *PmTNF* in the NCBI database ( https://www.ncbi.nlm.nih.gov/). Open reading frame (ORF) analysis was performed via the ORF Finder tool of NCBI ( https://www.ncbi.nlm.nih.gov/orffinder/). SMART was subsequently applied to analyze the detected PmTNF peptides, and a phylogenetic tree was constructed via MEGA X software. The protein molecular weight and theoretical isoelectric point (pI) were predicted via the ProtParam tool from ExPASy ( https://web.expasy.org/protparam/).

**Table:** **Table 1** Sequences of primers used in this study

Primer	Sequence (5′→3′)	Tm value (°C)	Application
PmTNF-5′-outer	GCCATTGTAAGATTGCCGTA	58.3	5′-RACE
PmTNF-5′-inner	CTTGCTATCACTCGCAGACG	61.0	5′-RACE
PmTNF-3′-outer	CAGCCAAGTCACATTTATCG	58.6	3′-RACE
PmTNF-3′-inner	AGTGACAGTTCCCGGTTTG	58.8	3′-RACE
PmTNF-ORF-F	CTTGCCGTCTGCGAGTGAT	60.0	Intermediate fragment
PmTNF-ORF-R	TCCAACAAACCGGGAACTG	58.0	Intermediate fragment
PmTNF -F	CCTACGGCAATCTTACAATGGCG	58.7	qRT-PCR
PmTNF -R	CTGATCCAACAAACCGGGAACTG	58.7	qRT-PCR
PmTRADD-F	TAAAAGATGGCGTAAACTAG	49.1	qRT-PCR
PmTRADD-R	CATTTGCCAGACAACTTCACTT	51.3	qRT-PCR
PmTRAF2-F	GGGAGTGAGGACAACGCAGT	60.3	qRT-PCR
PmTRAF2-R	GTGCCACTGGTCCCGAGT	61.1	qRT-PCR
PmNIK-F	ATCAGTGTCTCGCAGTTCGT	56.3	qRT-PCR
PmNIK-R	ATGGCTCTCCGTCCGAAC	57.8	qRT-PCR
PmIKK-F	TATTAAAGGCTCAGGCAGAGGTAT	54.9	qRT-PCR
PmIKK-R	TTGGAGTTGCTGATTACGGATT	53.8	qRT-PCR
PmNF-κB-F	AGAAGAGACAGGCCAAAGAGCA	58.5	qRT-PCR
PmNF-κB-R	AGAGAGAACAGGCGTGAGAAGC	59.4	qRT-PCR
PmTNF-RNAi-F	GCGTAATACGACTCACTATAGGG CTTGCCGTCTGCGAGTGAT	70.0	Interference
PmTNF-RNAi-R	GCGTAATACGACTCACTATAGGG TCCAACAAACCGGGAACTG	67.9	Interference
PmRFP-RNAi-F	GCGTAATACGACTCACTATAGGGCTGTCCCCCCAGTTCCAGTAC	70.6	Interference
PmRFP-RNAi-R	GCGTAATACGACTCACTATAGGGCGTTGTGGGAGGTGATGTCCAGCT	71.8	Interference
GAPDH-F	CACTCGCCAAGATAATCAACG	53.7	Reference gene
GAPDH-R	CCATTCCTGTCAACTTCCCAT	54.8	Reference gene

### Tissue sample collection

Normal tissues: Specific tissues such as the adductor muscle, mantle, hemolymph, hepatopancreas, gonads, and gills were collected from 10 pearl oysters and immediately preserved in liquid nitrogen. The hemolymph samples were centrifuged at 469 *g* for 5 min at 4°C, and the pellets, which represented the hemocytes, were preserved in an ultralow temperature freezer at –80°C.

Tissues after the immune stimulation experiment: A total of 240 pearl oysters were randomly assigned into three experimental groups, with each group comprising 80 individuals. Two of these groups received an intramuscular injection of 100 μL of 10 μg/mL lipopolysaccharide (LPS; Sigma, St Louis, USA) or Poly (I:C) (Sigma) dissolved in phosphate-buffered saline (PBS). In contrast, the control group was administered with an equivalent volume of PBS without any immunogen. After injection, gill tissues were harvested from a subset of six pearl oysters per group at various time points: 0, 3, 6, 12, 24, 48, 72, and 96 h.

Tissues after nucleus insertion surgery: Seventy pearl oysters underwent nucleus insertion at the cultivation base of Guangdong Ocean University, which is located in Zhanjiang, Guangdong Province, China. The surgery was performed by a seasoned technician following the established protocols detailed in a prior investigation [21]. Gill tissues from these oysters were collected at selected time intervals after insertion: 0, 6, 12, 24, 48, 72, and 96 h after insertion.

### RNA extraction and cDNA synthesis

Total RNA was extracted from the tissue samples via TRIzol reagent (Thermo Fisher Scientific, Waltham, USA). Single-strand cDNAs were synthesized with a PrimeScript ^TM^ first-strand cDNA synthesis kit (Takara Bio) and used as templates for quantitative real-time PCR (qRT-PCR) analysis.

### qRT-PCR analysis

Gene expression levels were measured via a LightCycler 96 real-time PCR system (Roche, Basel, Switzerland) with SYBR ^®^ Select Master Mix (Takara Bio) as previously described [22]. The reaction system included 0.4 μL of cDNA template, 0.4 μL of forward primer, 0.4 μL of reverse primer, and 3.8 μL of double-distilled water to bring the total volume to 10 μL. Three replicate experiments were performed for each sample. The reaction procedure was as follows: 95°C predenaturation for 5 min; 95°C for 15 s; and 60°C for 1 min for 40 cycles. The relative expression levels of the target genes were determined via the 2 ^–ΔΔ *CT*^ method. *GAPDH* was used as a reference gene.

### PmTNF gene silencing by RNA interference (RNAi)

RNAi were carried out as previously described [23]. Briefly, PCR products were amplified through the use of specific primers and subsequently purified via EasyPure Quick Gel Extraction Kits (Thermo Fisher Scientific). The primer sequences specifically designed for the amplification of both *PmTNF* and the red fluorescent protein ( *RFP*) gene are listed in Table 1. For the synthesis of double-stranded RNA (dsRNA) from the PCR products, the T7 High Efficiency Transcription Kit (TransGen Biotech, Beijing, China) was used. The dsRNA was then refined via EasyPure RNA Purification Kits (TransGen Biotech). The next step involved diluting each dsRNA preparation to a concentration of 600 μg/mL with RNase-free water.

To conduct the *PmTNF* RNAi experiments, 120 healthy pearl oysters were selected and allocated into three groups, each comprising 40 individuals. A distinct treatment was administered to each group via injection: one group received 100 μL of concentrated dsRNA- *PmTNF*, another was given an equal volume of diluted dsRNA-RFP to serve as a negative control, and the third group was injected with 100 μL of DEPC water to act as a blank control. Subsequent to the injections, gill tissues were collected from eight oysters per group at prescheduled intervals of 24, 48, 72, and 96 h.

For the *PmTNF* RNAi assay during nucleus insertion, 60 healthy pearl oysters were selected and evenly divided into three experimental groups, with 20 oysters per group. Each group was injected with 100 μL of a specified solution: diluted *dsRNA-PmTNF*, which served as the experimental treatment; diluted *dsRNA-RFP*, which served as the negative control; or 100 μL of DEPC water, which served as the blank control. Next, each oyster underwent nucleus insertion surgery. Gill tissues from eight pearl oysters per treatment group were collected and frozen in liquid nitrogen at 24 and 48 h after nucleus insertion. Following RNA extraction, the mRNA expression of tumor necrosis factor receptor-associated death domain protein (TRADD), tumor necrosis factor receptor-associated factor 2 (TRAF2), IκB kinase (IKK), NF-κB-inducing kinase (NIK), and nuclear factor kappa-B (NF-κB) was assessed via qRT-PCR, as described above. The sequences of the primers used to detect these transcripts are displayed in Table 1.

### Production of the rPmTNF protein

The cDNA fragment encoding the extracellular region of PmTNF, spanning positions 105-750, as depicted, was synthesized by HuaBio (Hangzhou, China). The cDNA fragment was subsequently cloned and inserted into the pET28a expression vector. The recombinant plasmid pET28a *-PmTNF* was transferred into Rosetta (DE3) competent cells, and the transformed bacteria were chosen and cultured in lysogeny broth (LB) media supplemented with 100 μg/mL kanamycin at 37°C until they reached an optical density of 0.6–0.8. The expression of the recombinant protein was subsequently induced by the addition of 1 mM isopropylbeta-D-thiogalactopyranoside (IPTG) for 4 h at 37°C. Following this induction period, the bacterial cells were collected by centrifugation at 4°C and then subjected to sonication while being maintained on ice to ensure cell lysis. The target protein was purified via HisTrap affinity columns. The purity and molecular weight of the resulting proteins were analyzed via SDS-PAGE. The purified rPmTNF protein (1 mg/mL) was stored at –80°C.

### Treatment with the rPmTNF protein and biochemical assays

The assay was performed as described by [ 14, 15] with some modifications. rPmTNF was diluted to 100 μg/mL with PBS. Ninety healthy pearl oysters were selected and evenly distributed into three experimental groups, with thirty oysters per group. Two groups were injected with 100 μL of diluted rPmTNF and 100 μL of PBS. Next, all the oysters underwent nucleus implantation. The control group underwent nucleus insertion only, without rPmTNF or PBS treatment. At 6, 12, and 24 h after injection and/or nucleus insertion, gill tissues from eight pearl oysters per treatment group were collected and frozen in liquid nitrogen.

A portion of each gill tissue sample was divided and homogenized in ice-cold physiological saline solution (w:v = 1:9) in addition to being utilized for total RNA production and qRT-PCR analysis and then centrifuged at 885 *g* at 4°C for 10 min. The supernatant was carefully collected for subsequent biochemical examination, and its crude protein content was determined by Coomassie Brilliant Blue G250 assay. Antioxidant enzymes, including superoxide dismutase (SOD), glutathione (GSH), and peroxidase (POD), as well as the total antioxidant capacity (T-AOC) of the extract were measured via the corresponding assay kits provided by Nanjing Jiancheng Institute of Bioengineering (Nanjing, China) following the manufacturer’s instructions.

### Statistical analysis

All experimental data were analyzed by Student’s *t* test and one-way ANOVA via SPSS 26.0 software (IBM, Armonk, USA). *P* < 0.05 was considered statistically significant.

## Results

### Cloning and sequence analysis of *PmTNF*

**Figure 2:**
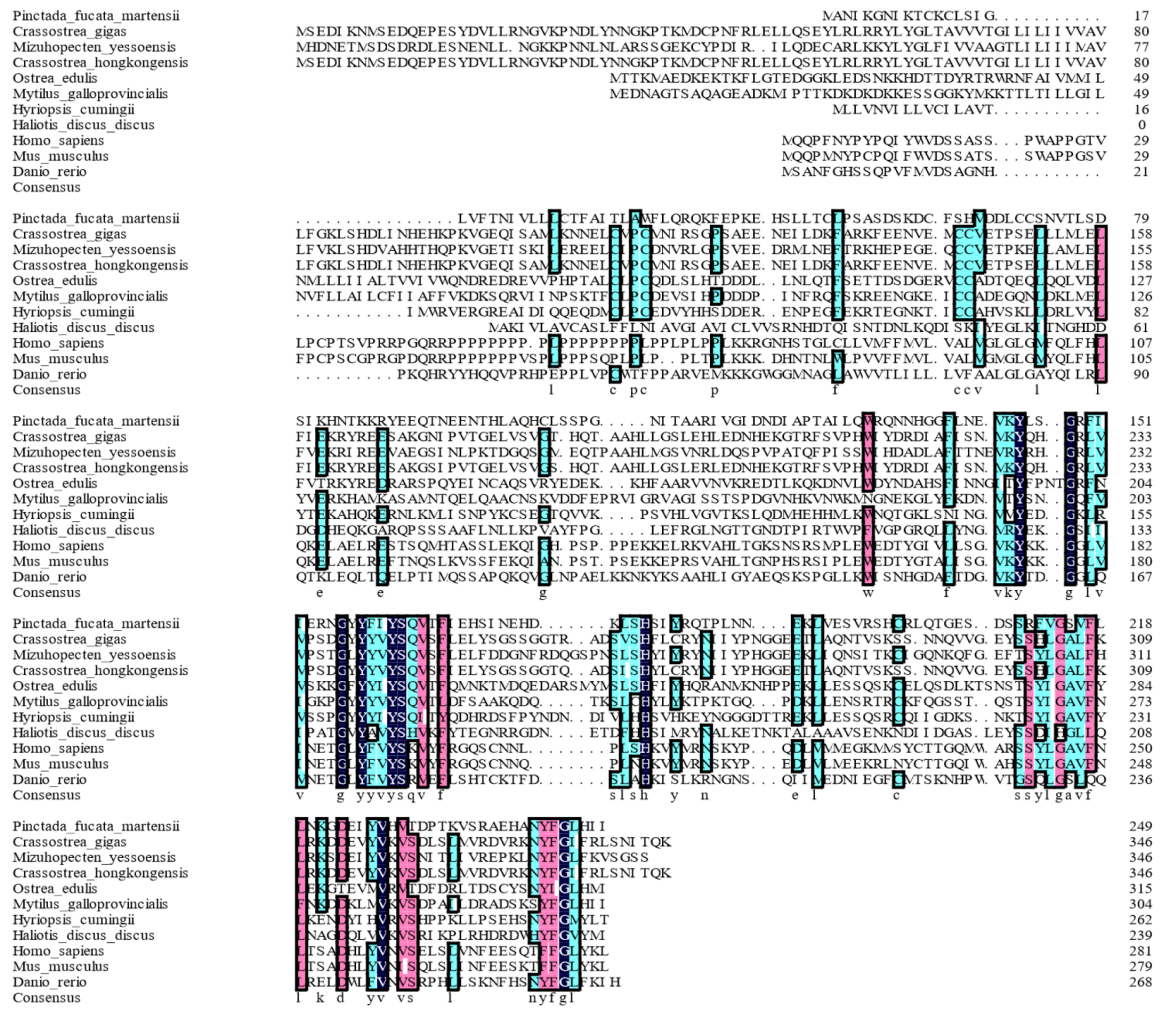
Figure 2 Amino acid multiple sequence alignment of PmTNF with TNF homologs from other species Dark blue, coincident amino acids; pink, similarity > 75%; turquoise, similarity > 50%. For each species, the GenBank accession numbers of the aligned sequences were as follows: Crassostrea gigas (XP_011450585.2), Crassostrea hongkongensis (APC65296.1), Mizuhopecten yessoensis (XP_021340408.1), Ostrea edulis (AFJ91808.1), Mytilus galloprovincialis (VDI20184.1), Hyriopsis cumingii (QJF53972.1), Haliotis discus discus (EU863217.1), Homo sapiens (NP_000630.1), Mus musculus (NP_034307.1), and Danio rerio (NP_001036166.1).

**Figure 3:**
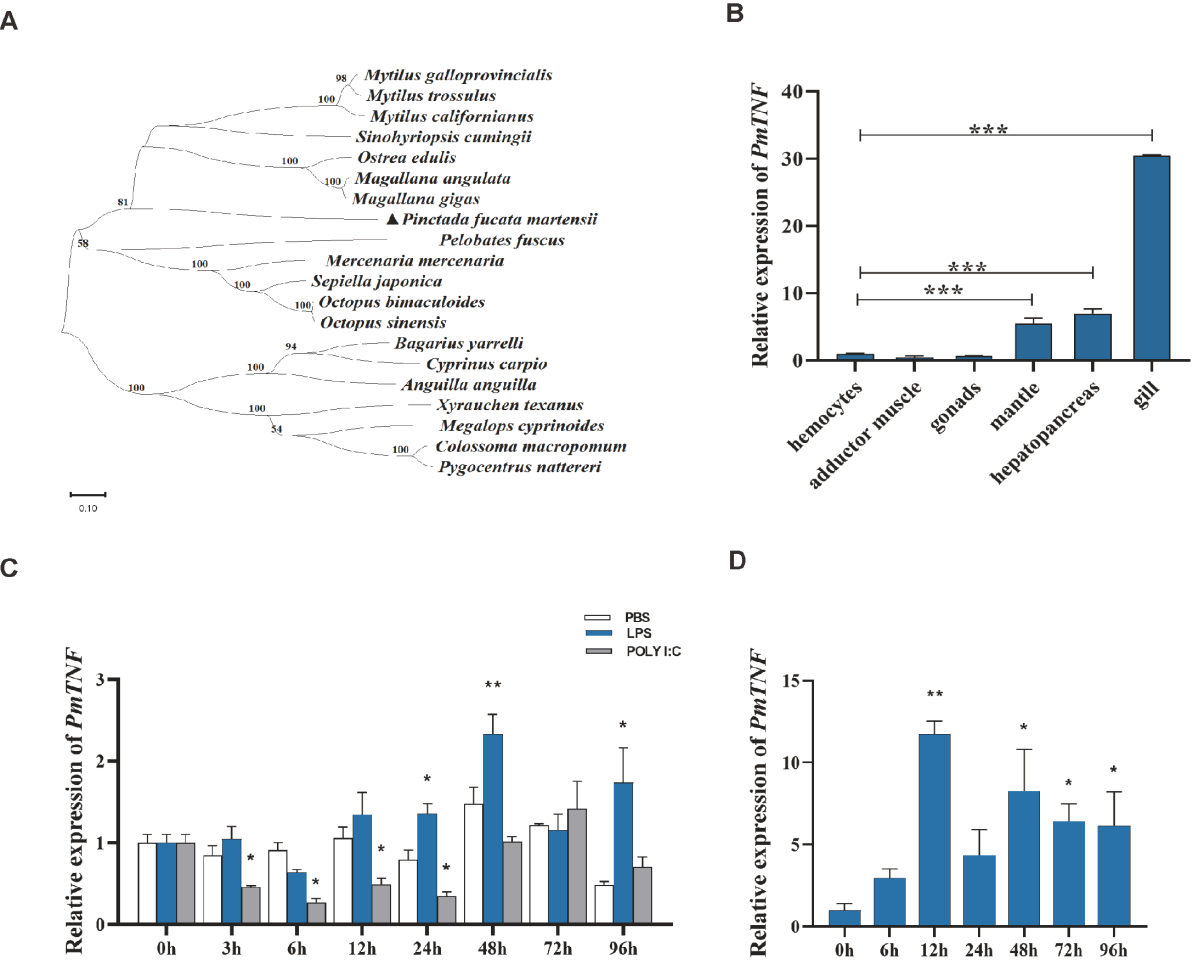
Figure 3 The evolutionary tree of TNF and its expression profile (A) Unroot neighbor-joining (NJ) phylogeny of TNF proteins from various vertebrate and invertebrate organisms. The GenBank accession numbers of the genes used for constructing the tree are provided in the supplementary data. (B) Constitutive gene expression of PmTNF in different pearl oyster tissues. Mean values with asterisks are significantly different according to one-way ANOVA. The vertical bars represent the standard deviation (***P < 0.001). (C) mRNA expression of PmTNF in pearl oyster gills after LPS and Poly (I:C) challenge. (D) mRNA expression of PmTNF in pearl oyster gills after nucleus insertion surgery. Mean values with asterisks are significantly different, as assessed by t tests. The vertical bars represent the standard deviation (*P < 0.05, **P < 0.01).

Partial cDNAs encoding a candidate TNF homolog were cloned from a *P* . *f*. *martensii* cDNA library via PCR (GenBank ID: OQ889552). The resulting PCR products were sequenced, and the full-length PmTNF cDNA consists of 1 413 bp, which contains an open reading frame of 750 bp, a 5′UTR of 76 bp and a 3′UTR of 587 bp, including a 33-bp poly(A) tail. The predicted ORF encodes a polypeptide of 249 amino acids with a putative molecular weight of 28.26 kDa and a predicted pI of 7.63. The deduced PmTNF protein encompasses a transmembrane domain and a THD. Additionally, multiple sequence alignments revealed that the deduced amino acid sequence of PmTNF has relatively low resemblance to the sequences of its counterparts in other species ( Figure 2). The corresponding phylogenetic tree, which was constructed via MEGA software via the neighbor joining (NJ) method, revealed that the TNF alignment product has a monophyletic interaction with high bootstrap support in the phylogenetic tree ( Figure 3A). One is a result of mollusca (and a chordata, amphibia), and the other is a result of chordata (pisces). In the former group, the cephalopods cluster together with relatively low node support.

### Expression pattern of PmTNF

The analysis of *PmTNF* expression via qRT-PCR revealed constitutive expression in all the tested oyster tissues. These relative expression levels were compared with those in hemocytes as a reference. The expression of *PmTNF* mRNA in tissues such as the gills, hepatopancreas, and mantle was greater than that in hemocytes. Furthermore, the gill tissues presented the highest expression, reaching levels approximately 30 times greater than those in hemocytes ( Figure 3 B).

### Expression profile of PmTNF in response to stress induced by immune challenge or nucleus insertion surgery

LPS and poly (I:C) are PAMP molecules recognized by innate receptors that trigger immune responses in the host. To test whether *PmTNF* expression is influenced by microbial stimulus, we exposed live pearl oysters to LPS or Poly (I:C) and monitored *PmTNF* expression levels in gills after treatment. Compared with that in control oysters treated with PBS, LPS induced a significant increase in *PmTNF* expression by 24 h, which peaked at 48 h. This increase vanished by 72 h, but *PmTNF* expression increased again at 96 h. In contrast, exposure to Poly (I:C) significantly downregulated *PmTNF* expression just after 3 h. This decrease, compared with that in the PBS-treated groups, persisted until 48 h, after which baseline *PmTNF* expression was restored ( Figure 3 C).

Upon stress caused by the surgical procedure consisting of nucleus insertion, *PmTNF* expression in gills was significantly upregulated from 12 h onward, with a transient decrease in expression at 24 h compared with that in the control group and persistent upregulation at later time points, between 48 and 96 h ( Figure 3D).

### Effect of *PmTNF* silencing on the expressions of immune-related genes in the NF-κB signaling pathway

**Figure 4:**
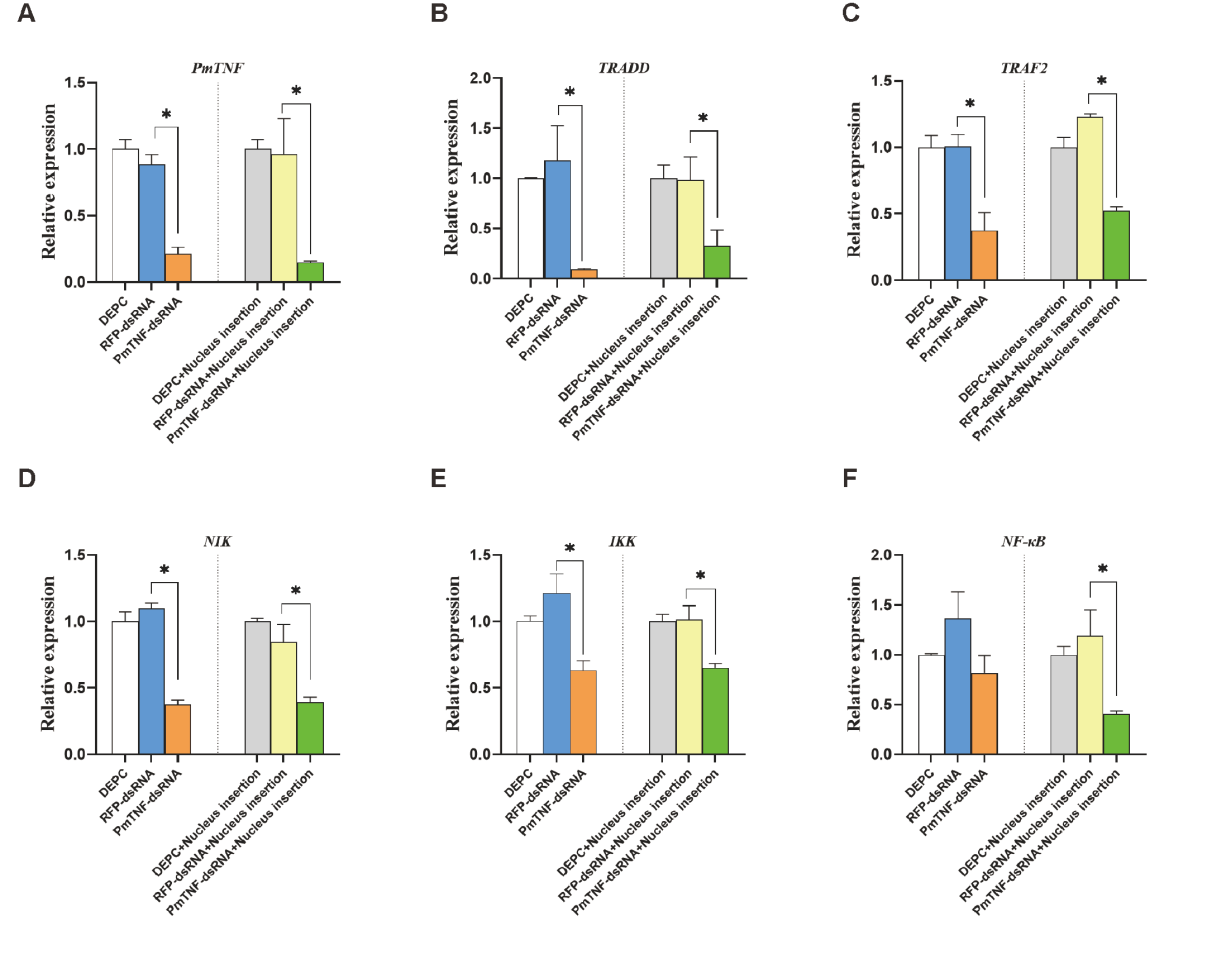
Figure 4 Assessment of the regulation of genes from the NF-κB signaling pathway by *PmTNF* via RNA interference Gene expression in gills after PmTNF silencing or PmTNF silencing and nucleus insertion at 24 h. Mean values with asterisks are significantly different, as assessed by t tests (*P < 0.05). (A) PmTNF silencing, with or without nuclear intervention at 24 h, significantly downregulates PmTNF expression. (B) PmTNF silencing significantly downregulates TRADD expression. (C) PmTNF silencing significantly downregulates TRAF2 expression. (D) PmTNF silencing significantly downregulates NIK expression. (E) PmTNF silencing significantly downregulates IKK expression. (F) PmTNF silencing does not affect NF-κB activity, but nuclear intervention following TNF silencing significantly downregulates NF-κB activity.

**Figure 5:**
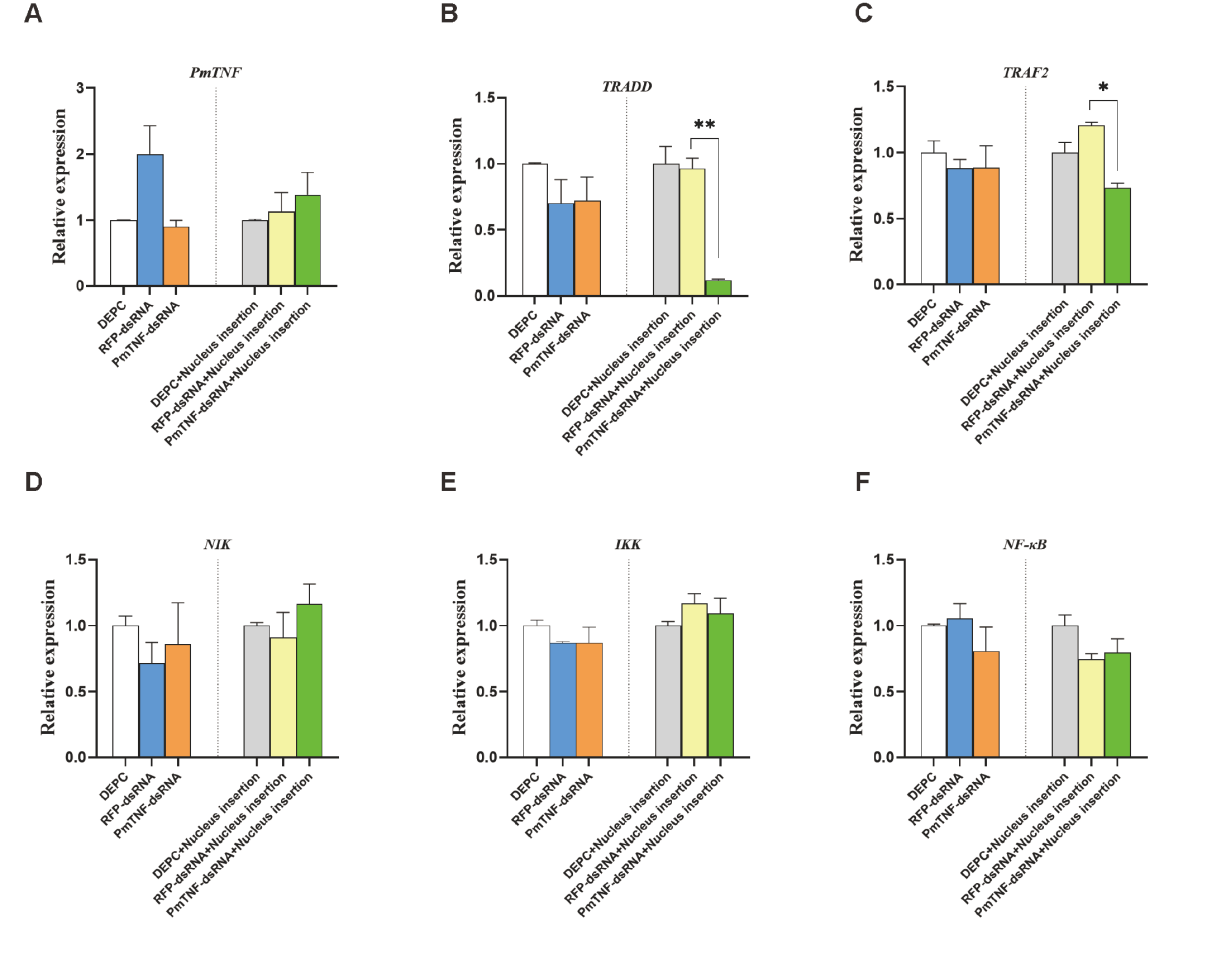
Figure 5 Assessment of the regulation of genes from the NF-κB signaling pathway by *PmTNF* via RNA interference Gene expression in gills after PmTNF silencing or PmTNF silencing and nucleus insertion at 48 h. Mean values with asterisks are significantly different, as assessed by t tests (*P < 0.05, **P < 0.01). (A) Silencing of PmTNF alone or with nucleus insertion at 48 h had no significant effect on expression levels. (B) TRADD levels were unaffected by PmTNF silencing, but were significantly downregulated after nucleus interference following PmTNF silencing. (C) TRAF2 levels were unchanged by PmTNF silencing, but significantly downregulated with subsequent nuclear interference. (D) NIK levels were not significantly changed by PmTNF silencing or nucleus insertion at 48 h. (E) IKK levels remained stable regardless of PmTNF silencing or nucleus insertion at 48 h. (F) NF-κB activity was not significantly altered by PmTNF silencing or nucleus insertion at 48 h.

To test whether *PmTNF* expression influences the NF-κB signaling pathway, we assessed the effect of *PmTNF* silencing on the expressions of genes in this pathway. To this end, we synthesized *dsRNA-PmTNF* RNAi specific for *PmTNF* and used *dsRNA-RFP* RNAi specific for RFP as a negative control. Compared with no treatment, the injection of *dsRNA-PmTNF* efficiently reduced *PmTNF* mRNA level in the gills at 24 h ( Figure 4). This silencing was transient and did not persist beyond 24 h, and 48 h of silencing hardly occurred. The stimulus effect may be even greater with the insertion of nuclear treatments ( Figure 5). Moreover, the decrease in *PmTNF* mRNA expression ( Figure 4A) was correlated with a significant reduction in *TRADD*, *TRAF2*, *NIK*, and *IKK* expression compared with that in the negative control group ( *P* < 0.05; Figure 4B–E). Thus, *PmTNF* influences the expressions of genes downstream of the NF-κB signaling pathway, with the exception of *NF-κB* itself.

These results prompted us to investigate whether *PmTNF* silencing during nucleus insertion could counteract the upregulation of *PmTNF* and therefore downregulate the expression levels of genes within the NF-κB signaling pathway. Pretreatment of the pearl oysters with *dsRNA-PmTNF* before nucleus insertion led to a rapid reduction in *PmTNF* mRNA level and transient downregulation of *TRADD*, *TRAF2* , *NIK*, *IKK*, and *NF-κB* at the 24-h mark ( *P* < 0.05; Figure 4B–E). These results suggest the possible regulation of the NF-κB signaling pathway by *PmTNF* during surgical stress.

### Effect of rPmTNF on the expressions of immune-related genes in the NF-κB signaling pathway during nuclear insertion

**Figure 6:**
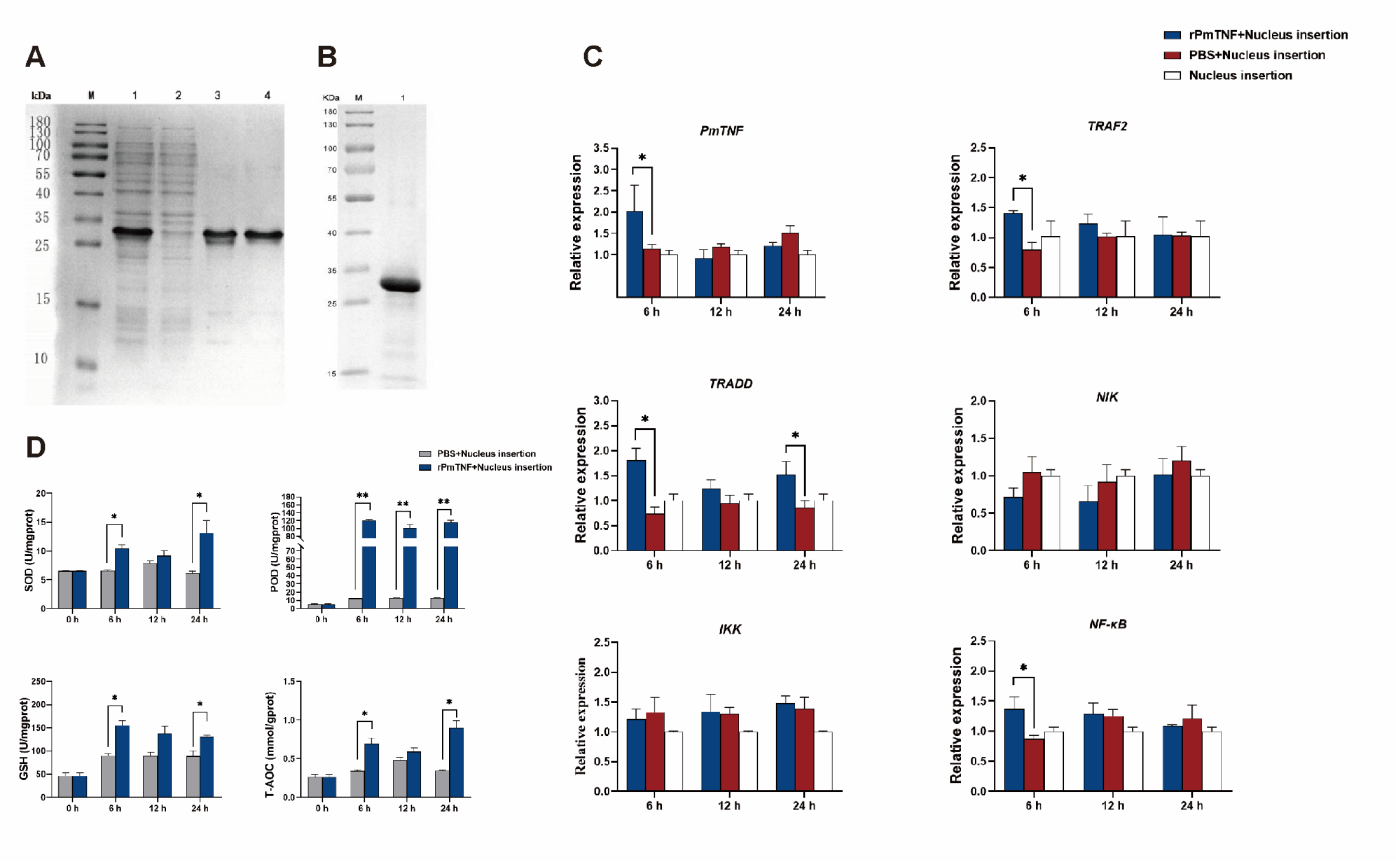
Figure 6 Prokaryotic expression of PmTNF and enzyme activity after PmTNF injection, as well as the expressions of NF-κB signaling pathway-related genes (A) Characterization of rPmTNF by SDS-PAGE analysis. Lane M: marker; Lane 1: full protein extract; Lane 2: flow through; Lanes 3--4: eluted proteins (two replicates). (B) Lane M: marker; Lane 1: protein concentrate sample. (C) Effect of rPmTNF on the expressions of genes involved in the NF-κB signaling pathway during nucleus insertion. Gene expression in the gills of pearl oysters treated with rPmTNF or PBS or left untreated and subsequent nucleus insertion. Mean values with asterisks are significantly different, as determined by t tests (*P < 0.05). (D) Effects of rPmTNF on the activities of SOD, POD, and GSH and on total antioxidant activity (T-AOC) in gills after nucleus insertion. Pearl oysters were treated with rPmTNF or PBS before nucleus insertion. The bar charts show the antioxidant activities of SOD, POD, and GSH, as well as T-AOC. Mean values with asterisks are significantly different, as determined by t tests. Significant differences are shown as * P < 0.05; **P < 0.01.

SDS-PAGE revealed that the purified rPmTNF protein constituted a major band at 28 kDa, which corresponds to the expected size of PmTNF ( Figure 6A). The concentrated protein was also expressed in a 28 kDa band ( Figure 6B). To study the effect of the rPmTNF protein *in vivo*, we tested the effect of rPmTNF on the expressions of genes related to the NF-κB signaling pathway during nuclear insertion. Challenge with rPmTNF induced the upregulation of *PmTNF* itself and most genes involved in the NF-κB pathway in gills by 6 h ( *P* < 0.05). However, the presence of rPmTNF did not influence *NIK* or *IKK* expression. The changes observed at 6 h were transient and were no longer visible at 12 h and beyond, except *TRADD* mRNA which displayed an s wave of upregulation at 24 h ( Figure 6C).

### Effect of rPmTNF on the activity of enzymes in the antioxidant system during nucleus insertion

The enzymatic activities of SOD, POD, and GSH, as well as the T-AOC, were compared after pearl oysters received or did not receive an injection of rPmTNF into the adductor muscle and subsequently underwent nucleus insertion. As shown in Figure 6D, at 6 h and 24 h after rPmTNF treatment and nucleus insertion, the activities of SOD, GSH and T-AOC in the gills were significantly greater in the treatment group than in the control group. However, no significant difference in SOD, GSH or T-AOC activity was detected at 12 h, suggesting that rPmTNF induced two waves of increased antioxidant activity. Interestingly, POD activity was significantly increased at 6 h and was maintained at 12 and 24 h after rPmTNF treatment and nucleus insertion.

## Discussion

Tumor necrosis factor (TNF) superfamily members are multipotent cytokines that induce a wide range of cellular responses in vertebrates, including cell proliferation, immune regulation, inflammation, cell death and apoptosis [ 3, 24]. Several molluscan species have also been shown to contain *TNF* genes, which are important for immunological reactions to bacterial and viral invasion [ 12, 13, 15]. In this study, *PmTNF* was cloned from the pearl oyster *P*. *f*. *martensii*. The ORF of *PmTNF* encompasses 750 bp, which corresponds to a polypeptide that is 249 amino acids in length. As reported for TNF family proteins in other species, the predicted PmTNF protein contains typical TNF and transmembrane domains, suggesting that it represents a novel member of the TNF superfamily. According to phylogenetic analysis, the closest homologous TNF sequences to those of PmTNF segregated into two branches, and PmTNF clustered with the mollusks’ homologs, indicating that PmTNF represents a bona fide TNF ortholog of genes previously described in these other species.

We detected *PmTNF* expression in the hepatopancreas, gills, and mantle in the natural state. The hepatopancreas and gills are acknowledged as primary immune organs in shellfish and are crucial for the host’s defense against pathogens. They play a pivotal role in responding to external stimuli, initiating inflammatory reactions, and assisting in repair following injury [ 25, 26]. The hepatopancreas of bivalves functions dually as a digestive and metabolic center, combining the roles of higher animal livers and digestive glands. Moreover, it serves as a crucial site for the immune response and detoxification. When food traverses through the gills into an organism’s digestive and metabolic system, the hepatopancreas plays a pivotal role in detoxifying and metabolizing harmful substances [27]. The gills, vital immune organs in mollusks, also serve as filter-feeding mechanisms that directly interface with the external environment. They perform a dual function: filtering bacteria and noxious elements from the water while concurrently preventing the ingress of bacteria and toxins into the organism [ 28, 29]. The outer mantle membranous rim area directly interfaces with the external environment and plays a significant role in the immune defense system of shellfish [30]. Compared with that in other tissues, *PmTNF-α* is highly expressed in pearl oyster gills, in accordance with previous observations of AbTNF-α in disk abalone [12] and of *Cg*TNF-1 and *Cg*TNF-2 in Pacific oyster [ 14, 15]. However, the highest expression level of *Ch*TNF [13] was observed in the muscle. It can be postulated that high expression in gill tissues, which play an important immune role, indicates a potential protective function of *PmTNF* .

PAMPs are recognized immediately and efficiently as “non-self” signals, activating the innate reaction [ 31, 32]. LPS constitutes a principal structural element of the majority of gram-negative bacterial species, which most likely stimulate innate immunity [33], whereas Poly (I:C) is a synthetic viral dsRNA that is extensively used to mimic viral infections [34]. In zebrafish [35] and grass carp [36], LPS upregulates TNF expression. Like in these teleosts, LPS increased *PmTNF* expression, whereas Poly (I:C) had the opposite effect. Current models in mollusks indicate that LPS induces TNF transcription by activating LPS-induced TNF-α factor (LITAF), which is immediately upregulated after challenge [ 33, 37]. Similarly, in disk abalone, AbTNF-α gene expression increased significantly in the gills after LPS exposure, peaking between 24 and 48 h after exposure [12], and *Cg*TNF-2 and *Ch*TNF expression in hemocytes significantly increased upon LPS challenge [ 13, 15]. Interestingly, in the shrimp *Marsupenaeus japonicus*, *Mj*TNF expression was not induced upon exposure to LPS. Rather, a significant increase in *Mj*TNF transcript abundance was observed upon exposure to Poly (I:C) [10]. The positive regulation of two TNF-α family members after 12 h of exposure to Poly (I:C) was also observed in snakehead [7]. These variable effects of LPS and Poly (I:C) on TNF expression suggest different mechanisms of TNF regulation and/or different TNF functions across species. LPS induces the production of TNF-α, and then newly synthesized TNF-α subsequently attaches to the TNF receptor to initiate a late NF-κB activation phase in an autocrine manner [38]. Moreover, NF-κB is crucial for Sendai virus and Poly(I:C) induction of IFNβ [39]. Therefore, the differential regulation of *PmTNF* expression by LPS and Poly (I:C), which mimic microbial infections, suggests that after immune stimulation, LPS can induce TNF-α, whereas Poly (I:C) can induce IFN. Furthermore, we found that the injury caused by nucleus insertion induced a significant peak in *PmTNF* upregulation at 12 h. Similarly, *PmTLR4* expression increased significantly after nucleus insertion [40]. These similar patterns suggest that surgery causes pathogenic infection of the host oyster and triggers an immune reaction [41]. Collectively, these data support the hypothesis that PmTNFs may be involved in immune responses against foreign bodies.

TNF, a proinflammatory cytokine, plays an essential and dominant role in the activation of the NF-κB pathway [42]. Upon induction by different stimuli, TNF binding to TNF receptors on the cell surface can activate the NF-κB signaling pathway [43]. Some TNF receptors have an intracellular domain termed the death domain, which recruits a number of adaptors, such as the TNFR-associated death domain (TRADD), to their cytoplasmic domains. TNF receptors lacking a death domain instead recruit TNFR-associated factor (TRAF) proteins such as TRAF2 through peptide motifs [44]. The resulting multimeric protein complexes are crucial regulators of cell fate. They lead to conformational changes in protein trimers and are polyubiquitinated. In turn, they induce the NF-κB pathway, which ultimately transactivates cytoprotective genes and facilitates cell survival [45]. TRAF2 can interact with NIk [46], but NIK cannot bind to TRADD, since the TRADD binding motif differs from that of the TRAF domain. TRADDs are involved in NF-κB induction downstream of TNF receptors, likely by interacting with TRAF2 [47]. Moreover, NF-κB bound to the inhibitor IκBs is liberated and translocated to the nucleus, where NF-κB can activate diverse target genes. This liberation occurs upon rapid Ikk-mediated phosphorylation and degradation of IκBs [48]. Thus, all the immune-related genes tested in this study have been implicated in NF-κB activation by TNF [46].

RNA interference is a powerful tool for analyzing the loss of function of genes *in vivo* and altering gene expression. In zebrafish, TNF-dsRNAs have been successfully used to reduce TNF-α mRNA and protein expression in virus-infected cells, accompanied by a decrease in NF-κB protein expression [49]. Additionally, a previous study demonstrated that in *P*. *f*. *martensii*, knockdown of *PmTNFR1* significantly downregulated *PmTNFR1* expression and suppressed downstream genes of the NF-κB signaling pathway [50]. Similarly, we found that *PmTNF-dsRNAs* could downregulate *PmTNF* expression, as well as downstream genes associated with the NF-κB signaling pathway in the immune response. Furthermore, *PmTNF* silencing combined with nuclear insertion inhibited the expressions of genes involved in NF-κB signaling. These findings suggest that *PmTNF* may participate in innate immune responses by activating the NF-κB pathway.

Previous studies have shown that rainbow trout leucocyte activity can be increased by human recombinant TNF-α [51]. At the molecular level, turbot recombinant TNF enhances IL-1β and TNF-α expressions in the goldfish *Carassius auratus L*. [52], and treatment with recombinant TNF-New protein significantly upregulated IL-1β expression in rock bream [8]. Furthermore, in grass carp, recombinant gcTNF-α can induce the phosphorylation of IκB and upregulate the expressions of TRAF1 and TRAF2 in a time-dependent manner [36]. To further study the biological activity of *PmTNF*, we produced a recombinant PmTNF protein. Oysters treated with this protein increased *PmTNF* mRNA expression and the expressions of several genes in the NF-κB signaling pathway but failed to induce the expression of *NIK* or *IKK*, suggesting that TNF may also activate NF-κB through an alternative pathway. These results further support the theory that *PmTNF* may function as a regulator of NF-κB activation.

Cell signaling plays a central role in the activation of immune cells, inflammatory responses, and pathogen clearance [53]. Cell signaling can induce or inhibit the expression or activity of specific enzymes, thereby regulating cellular metabolism and function [54]. Enzyme activity can activate signal transduction pathways and transcription factors [55], affecting the synthesis and secretion of cytokines, which are important messengers in the immune system, including IFNs, ILs, and TNFs, and they play a core role in immune regulation [53]. rPmTNF supplementation increased the activities of antioxidant enzymes, including SOD, POD, and GSH as well as the T-AOC in the gills of pearl oysters that underwent nucleus insertion. Antioxidant enzymes, such as SOD, are released to maintain oxidative balance and prevent tissue damage. These enzymes are produced during immune responses and inflammation, during which they prevent collateral cell toxicity by maintaining the oxidation/antioxidant balance in organisms [56]. In Pacific oysters receiving an injection of recombinant *Cg*TNF-2, lysozyme activity and serum nitric oxide levels, which represent antioxidant activity, increase significantly [15]. Similarly, turbot recombinant TNF induced significant nitric oxide production from phagocytes [52]. By modulating enzyme activity, the function of the immune system can be finely tuned, which is highly important for immune-related events. In this study, after injection of rPmTNF, the enzyme activity in the gills was significantly greater than that in the control group. These findings indicate that the abundance of immune factors in the gills was significantly greater in the treatment group than in the control group. Moreover, a rapid response was observed within 6 h, suggesting that exogenous injection can enhance the immune and antioxidant capabilities of the organism. Thus, the dual effects of rPmTNF on immune-related genes in the NF-κB signaling pathway and its antioxidant activity suggest that *PmTNF* plays a critical role in the pearl oyster immune system.

In summary, in this study a newly-identified *PmTNF* gene was successfully cloned and characterized from the pearl oyster *P*. *f*. *martensii*. *PmTNF* expression was constitutive in all the tested tissues, with high expression in the gills, where it could be significantly induced by LPS, Poly (I:C), and nucleus insertion. Knockdown of *PmTNF* by interfering RNA inhibited *PmTNF* expression and downregulated immune-related genes of the NF-κB signaling pathway at steady state and during nucleus insertion. Conversely, injection of rPmTNF during nucleus insertion upregulated the expressions of *PmTNF* and several genes in the NF-κB pathway. Moreover, rPmTNF enhanced the activities of the antioxidant enzymes SOD, POD, and GSH, as well as the T-AOC. These findings provide new insights into the role of *PmTNF* in the defense systems of pearl oysters through NF-κB signaling and antioxidant activity.

## Supporting information

supplement_file

## Supplementary Data

Supplementary data is available at *Acta Biochimica et Biophysica Sinica* online.
