# Supplementary material for: Regulation of immune responses by a tumor necrosis factor in pearl oysters: insights from PmTNF gene expression and function : Characterization of novel TNF in pearl oyster
Source: Acta Biochim Biophys Sin (Shanghai). 2025 Feb 27;57(7):1081–92. doi: 10.3724/abbs.2025003 (PMC12367993; doi:10.3724/abbs.2025003)
Supplement: supplement_file [file supplement_file.docx]

1 acatggggaataccaagtgcgcacagaataagtaatattttattcatgaatttgctaacttagattggggagctatATGGCGAACATCAA

1. M A N I K

91 GGGAAATATAAAAACCTGCAAGTGCTTGTCAATCGGACTTGTGTTTACTAACATAGTCTTACTGCTTTGTACATTTGCTATTACTTTAGC

6 G N I K T C K C L S I G L V F T N I V L L L C T F A I T L A

181 TTGGTTCCTACAGCGACAGAAGTTCGAACCAAAAGAACATTCACTTTTGACTTGCTTGCCGTCTGCGAGTGATAGCAAGGACTGCTTTTC

36 W F L Q R Q K F E P K E H S L L T C L P S A S D S K D C F S

271 CCATGTTGATGATTTGTGCTGTAGCAATGTGACACTATCAGATTCTATTAAACATAACACCAAGAAAAGATATGAAGAACAGACCAATGA

66 H V D D L C C S N V T L S D S I K H N T K K R Y E E Q T N E

361 AGAAAATACACATTTAGCTCAGCATTGTCTCTCCTCACCAGGGAATATAACGGCAGCAAGGATTGTCGGAATAGACAATGACATTGCACC

96 E N T H L A Q H C L S S P G N I T A A R I V G I D N D I A P

451 TACGGCAATCTTACAATGGCGCCAGAACAACCATGGAGGATTCCTCAATGAAGTCAAATACTTATCAGGAAGATTCATCATTGAAAGGAA

126 T A I L Q W R Q N N H G G F L N E V K Y L S G R F I I E R N

541 TGGATATTATTTCATATACAGCCAAGTCACATTTATCGAACACTCCATAAACGAACATGACAAGCTGTCACACTCCATTTACAGACAAAC

156 G Y Y F I Y S Q V T F I E H S I N E H D K L S H S I Y R Q T

631 TCCATTGAACAATGAAAAACTTGTTGAGAGTGTTCGATCACACTGTAGACTACAGACAGGAGAGAGTGACAGTTCCCGGTTTGTTGGATC

186 P L N N E K L V E S V R S H C R L Q T G E S D S S R F V G S

721 AGTCTTTCTCTTAAACAAAGGTGATGAAATATACGTACATGTTACTGATCCTACTAAAGTATCACGTGCCGAACACGCTAATTATTTCGG

216 V F L L N K G D E I Y V H V T D P T K V S R A E H A N Y F G

811 TCTTCATATTATATAGactaaccttgtttatggtagaggttggaagtaaaatttagaagtatgctacttagttgagcacctcggtaattg

246 L H I I *

901 agcacctcggttgcagtacaatttttgtaatattttttatcatgaaaatcaaatgtatgcctcgagactgacacttttttttcaaatgcc

991 ataataatattgagggatcgccaaatgtcccatacttgctggcagactttttgactgtgatatcaaaaggaatggccaatcaaactgtgc

1081 tttaacgggccattgtttatgacgtcatgatgtataagtttgccaaccggatattcggcgatcacttatttcatgatagtacttttgtga

1171 attgcaatagtgatatgcttattttgtaaatattattttcctttttcaattttgcaatgattttatggaattgcgagtgttttgatttca

1261 tacaatggtccatatcctacatgtaattgtagcaccagtgtttggactctttagagtttttgttttattataactagttatagaagttca

1351 tgtttgttcatattttacttgttattcaagaaaaaaaaaaaaaaaaaaaaaaaaaaaaaaaaa
